# Ruptured solitary fibrous tumor of the pleura with hemothorax: a case report

**DOI:** 10.1186/s40792-024-02044-z

**Published:** 2024-10-23

**Authors:** Hiroaki Komatsu, Nao Furukawa, Kosuke Imamoto, Kazunori Okabe

**Affiliations:** Departments of Thoracic Surgery, Bell-Land General Hospital, 500-3, Higashiyama, Naka-Ku, Sakai-Shi, Osaka 599-8247 Japan

**Keywords:** Solitary fibrous tumor, Surgery, Rupture, Hemothorax

## Abstract

**Background:**

The majority of the patients with a solitary fibrous tumor (SFT) of the pleura are asymptomatic, and rupture of an SFT with hemothorax is rare.

**Case presentation:**

A 48-year-old man was taken by ambulance to our hospital because of sudden onset of left chest pain. Two months before the referral, a tumor was detected in the left upper lobe of the lung by screening computed tomography at another hospital, and further observation was recommended, because the tumor was suspected to be benign. Our contrast-enhanced computed tomography analysis of the chest revealed a solid tumor (5 cm in diameter) with an irregular enhancement effect close to the pericardium and pleural effusion in the left thoracic cavity. Pleural effusion was not detected in the previous imaging analysis. CT number of the pleural effusion was 40 HU, and the pleural effusion was suspected to be hematogenous. Therefore, rupture of the tumor with bleeding was suspected as the cause of the effusion because of the sudden onset. Preoperative diagnosis was a mediastinal tumor, such as a teratoma, because the tumor was close to the pericardium. Thoracoscopic surgery was performed with the patient in the right lateral decubitus position; bloody pleural effusion was observed and drained. The tumor originated from the visceral pleura of the left upper lobe of the lung and was resected with a surgical stapler. Macroscopic analyses of the resected tumor indicated that bleeding were caused by the rupture of the tumor at the defect of the capsule wall. The operation took 63 min. The postoperative pathological diagnosis was a benign SFT. Hemorrhage was observed just under the capsule wall of the tumor. The postoperative course of the patient was uneventful, and he was discharged 2 days after surgery.

**Conclusions:**

Even when an SFT is neither huge nor malignant, rupture can occur, and resection should be considered regardless of the size or malignant characteristics. After an SFT rupture, careful follow-up is needed to monitor for the intrathoracic recurrence or dissemination of the tumor.

## Background

Solitary fibrous tumor (SFT) of the pleura is a rare tumor of mesenchymal origin. The majority of patients are asymptomatic [1–3]. Symptoms are primarily due to compression caused by large tumors and include chest pain, cough, and dyspnea [3, 4]. Rupture of an SFT with hemothorax is very rare [1–8]. Here, we report a case of a patient with a ruptured SFT with hemothorax that underwent thoracoscopic resection.

## Case presentation

**Fig. 1:**
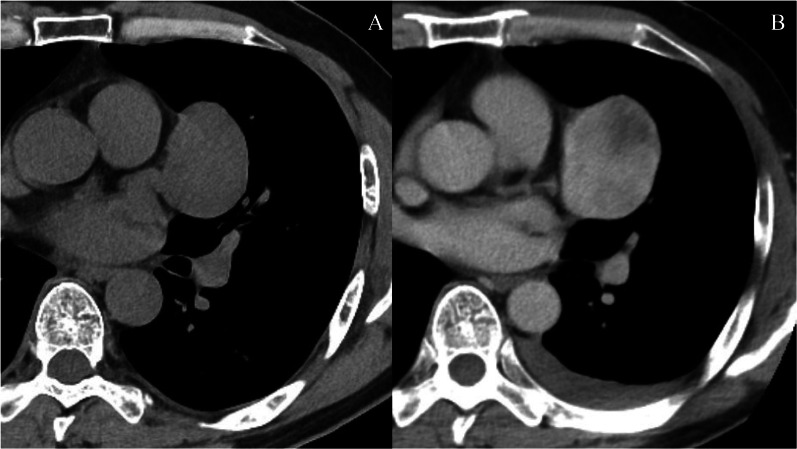
Chest CT images at the time of detection (**A**) and after tumor rupture (**B**). **A** Chest CT imaging showing a tumor in the left upper lobe of the lung. **B** Chest contrast-enhanced CT imaging showing a solid tumor (5 cm in diameter) close to the pericardium, and left pleural effusion. *CT* computed tomography

**Fig. 2:**
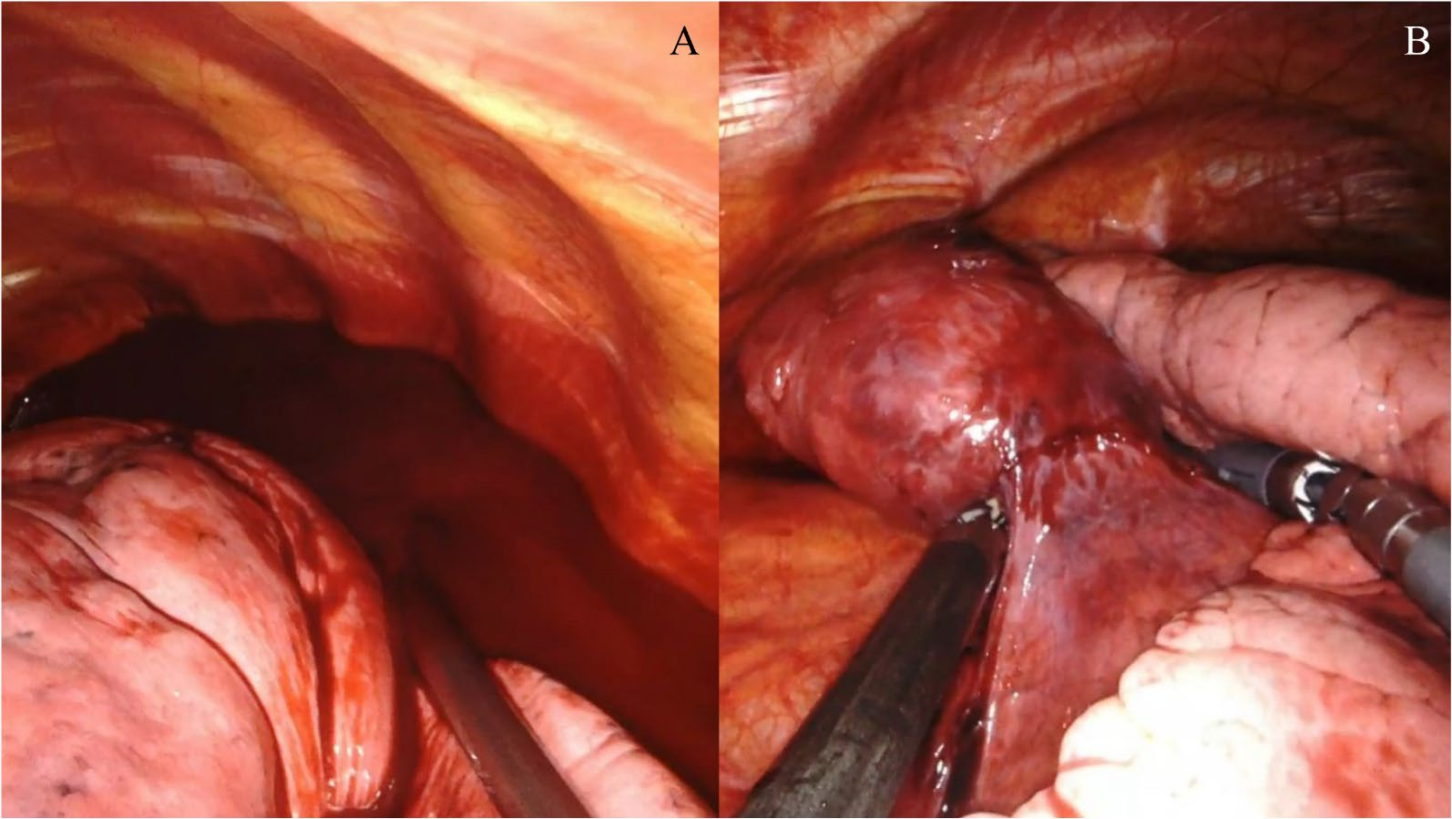
Surgical views of the tumor and hemothorax. **A** Surgical view showing bloody pleural effusion in the left thoracic cavity. **B** Surgical view showing the tumor origin in the visceral pleura in the left upper lobe of the lung

A 48-year-old man was taken by ambulance to our hospital because of sudden onset of left chest pain. He woke up with the chest pain in the early morning, and his symptom continued. Six hours after the sudden onset, he was taken to our hospital on suspicion of the ischemic heart disease. Two months before the referral, a tumor was detected in the left upper lobe of the lung by screening computed tomography (CT) at another hospital (Fig. 1A), and he was asymptomatic. Further observation was recommended and follow-up CT was planned after 6 months, because the tumor was suspected to be benign. At our hospital, contrast-enhanced CT revealed a solid tumor (5 cm in diameter) with an irregular enhancement effect close to the pericardium, and pleural effusion in the left thoracic cavity (Fig. 1B). Pleural effusion was not detected in the previous imaging analysis (conducted 2 months before). CT number of the pleural effusion was 40 HU, and the pleural effusion was suspected to be hematogenous. Therefore, rupture of the tumor with bleeding was suspected as the cause of the effusion because of the sudden onset. Preoperative diagnosis was a mediastinal tumor, such as a teratoma because of the higher frequency of rupture of these tumors. Thoracoscopic surgery was performed with the patient in the right lateral decubitus position, and bloody pleural effusion was observed and drained (Fig. 2A). The amount of sacked bloody pleural effusion was about 200 ml. The tumor was adhered to the pericardium, which was dissected. The tumor originated from the visceral pleura of the left upper lobe of the lung and was resected with a surgical stapler (Fig. 2B). Combined resection of the lung parenchyma was performed to secure the surgical margin.

**Fig. 3:**
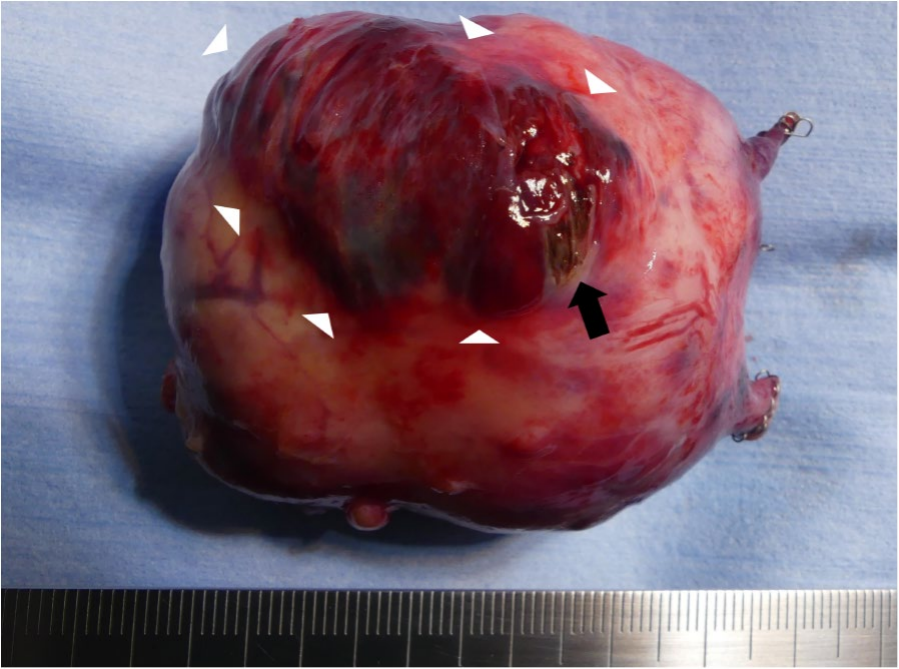
Macroscopic analysis of the tumor. Macroscopic analysis of the resected tumor showing hemorrhage just under the capsule wall of the tumor (arrowhead) and rupture of the tumor at the defect of the capsule wall (arrow)

**Fig. 4:**
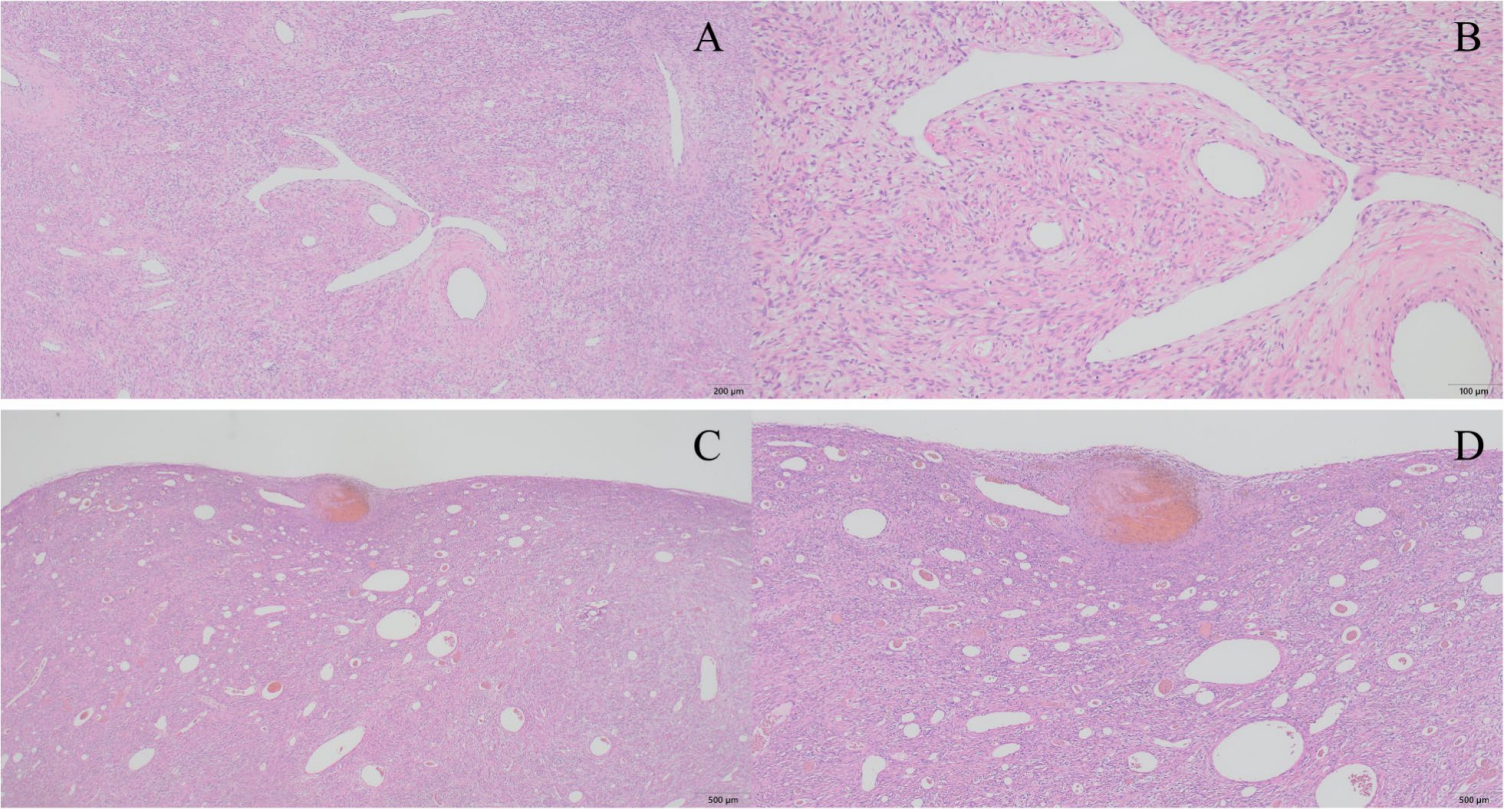
Microscopic analysis of the tumor (**A** low-magnification, **B** high-magnification). Microscopic analysis of the resected tumor showing a benign solitary fibrous tumor. Neither nuclear atypia nor necrosis was observed, and mitotic figures were rarely detected. Cellularity in the tumor was moderate (**C** low-magnification, **D** high-magnification). There were increased capillary growth and hemorrhage just under the capsule wall of the tumor

Macroscopic analysis of the resected tumor indicated that bleeding was caused by the rupture of the tumor at the defect of the capsule wall (Fig. 3). Hemorrhage was observed just under the capsule wall of the tumor (Fig. 3). The total operating time was 63 min. The postoperative pathological diagnosis was a benign SFT (Fig. 4A, B). Microscopic analysis of the resected tumor showed increased capillary growth and hemorrhage just under the capsule wall of the tumor (Fig. 4C, D). Neither nuclear atypia nor necrosis was observed, and mitotic figures were rarely detected. Cellularity in the tumor was moderate. The postoperative course of the patient was uneventful, and he was discharged 2 days after the surgery.

## Discussion

A review of SFT studies found eight cases of ruptured SFT with hemothorax [1–8]. The characteristics of these studies are summarized in Table 1. The median size of the ruptured tumors was 9.4 cm. Most patients had large tumors, whereas only one had a small, 3 cm-diameter tumor. Two tumors had malignant characteristics, according to the pathologic criteria cited in England et al. [9]. The tumor in our patient was benign and smaller than most tumors reported. Tumor rupture and hemothorax were reported to be caused by a rapid increase in tumor size [1], the rupture of blood vessel capillaries, or necrosis [4]. The tumor in our patient exhibited neither necrosis nor a rapid increase in size. The hemorrhage just under the tumor capsule wall was associated with the rupture of capillaries, which might have caused the rupture of the tumor. Even when an SFT is neither huge nor malignant, bleeding and tumor rupture can occur.

**Table 1:** Characteristics of previous reports of rupture of an SFT with hemothorax

Author	Age	Sex	Tumor size	Pathology
Tan et al. [1]	76	Woman	9.7	Benign
Shao et al. [2]	48	Woman	3	Benign
Negri et al. [3]	38	Woman	7	Benign
Leng et al. [4]	26	Man	18.2	Malignant
Kristensen et al. [5]	34	Man	11	Benign
Asai et al. [6]	31	Man	9	Benign
Morita et al. [7]	37	Man	7	Benign
Ikeda et al. [8]	71	Man	20	Malignant

Two of the eight patients described in the previous reports initially refused an operation, and an emergency operation was required immediately after tumor rupture [2, 4]. If our patient had undergone tumor resection upon detection, tumor rupture would have been avoided. The gold standard treatment for SFT remains surgery [10]. Following detection of a suspected SFT, resection should be considered regardless of tumor size or malignant characteristics.

Diagnosis of SFT of the pleura is challenging due to the lack of a specific radiographic appearance [1]. In our patient, the tumor was close to the pericardium, and a mediastinal tumor, such as a teratoma, was initially suspected because of the higher frequency of rupture of these tumors. The magnetic resonance imaging (MRI) should be considered preoperatively, because the MRI can help in differential diagnosis of SFT [11]. We performed thoracoscopic resection with the patient in the right lateral decubitus position. In contrast, most patients described in the previous studies underwent posterolateral thoracotomy because of the large size of the tumors. Whether the origin of the tumor is mediastinum, visceral or parietal pleura, or in the lung, thoracoscopic surgery with the patient in the lateral decubitus position is the most versatile for detecting the origin of the tumor and the bleeding; the hemostasis of bleeding; the wedge or anatomical resection of the lung; the resection of the mediastinal tumor; and the conversion to the thoracotomy.

The recurrence rate for benign SFT of the pleura is between 5 and 17% [10]. After tumor rupture, the intrathoracic recurrence or dissemination of the tumor is a concern. A patient who had undergone resection of a giant malignant SFT exhibited recurrence in the ipsilateral thoracic cavity 6 months after surgery [8]. Postoperative pleural dissemination of benign SFT has also been reported [12]. According to the 2020 World Health Organization classification, SFTs are mesenchymal tumors with intermediate biological behavior [13]. This suggests that SFTs cannot be wholly distinguished as benign or malignant. Whether a tumor is benign or malignant, careful follow-up is needed to detect a recurrence after SFT rupture.

## Conclusions

Even when an SFT is neither huge nor malignant, rupture can occur, and resection should be considered regardless of the size or malignant characteristics. After SFT rupture, careful follow-up is needed to monitor for intrathoracic recurrence or dissemination of the tumor.

## Data Availability

All data are available upon reasonable request.

## Abbreviations

SFT: Solitary fibrous tumor
CT: Computed tomography
